# Combinatorial Quantification of Multi-Features of Coda Waves in Temperature-Affected Concrete Beams

**DOI:** 10.3390/ma17092147

**Published:** 2024-05-03

**Authors:** Gang Zheng, Linzheng Song, Wenqi Xue, Zhiyu Zhang, Benniu Zhang

**Affiliations:** 1 State Key Laboratory of Mountain Bridge and Tunnel Engineering, Chongqing Jiaotong University, Chongqing 400074, China; songlz2022@163.com (L.S.); xuewq0101@foxmail.com (W.X.); 622220970057@mails.cqjtu.edu.cn (Z.Z.); benniuzhang@cqjtu.edu.cn (B.Z.); 2 School of Civil Engineering, Chongqing Jiaotong University, Chongqing 400074, China

**Keywords:** coda wave, signal multi-feature, concrete, temperature, structural health monitoring, signal processing

## Abstract

Coda waves are highly sensitive to changes in medium properties and can serve as a tool for structural health monitoring (SHM). However, high sensitivity also makes them susceptible to noise, leading to excessive dispersion of monitoring results. In this paper, a coda wave multi-feature extraction method is proposed, in which three parameters, the time shift, the time stretch, and the amplitude variation of the wave trains within the time window, are totally derived. These three parameters are each mapped to the temperature variations of concrete beams, and then combined together with their optimal weight coefficients to give a best-fitted temperature–multi-parameter relationship that has the smallest errors. Coda wave signals were collected from an ultrasonic experiment on concrete beams within an environmental temperature range of 14 °C~21 °C to verify the effectiveness of the proposed method. The results indicate that the combination of multi-features derived from coda wave signals to quantify the medium temperature is feasible. Compared to the relationship established by a single parameter, the goodness-of-fit is improved. During identification, the method effectively reduces the dispersion of identification errors and mitigates the impact of noise interference on structural state assessment. Both the identification accuracy and stability are improved by more than 50%, and the order of magnitude of the identification accuracy is improved from 1 °C to 0.1 °C.

## 1. Introduction

Concrete, as one of the most widely used building materials worldwide, has led to several safety issues of varying degrees in a large number of in-service concrete structures. Therefore, the monitoring and warning of its structural state through high-precision monitoring technology is an important means to prevent serious accidents.

Non-destructive testing (NDT) is favored by engineers, as it does not cause damage to the concrete structure itself. Various technologies such as strain gauges (SGs), electro-mechanical impedance (EMI), fiber Bragg grating (FBG), etc., which are based on different working principles and scopes, have been developed and widely used in SHM. For instance, traditional sensors such as SGs and thermocouples are technically mature and inexpensive, and can measure the localized state of a concrete structure; the optical properties of light in the fibers of FBG sensors show high sensitivity to multiple parameter changes in the structural state [1]; EMI monitors the state of the structure through the structural response under electromagnetic excitation [2]; and magnetic testing (MT) [3], radar wave detection (RWD) [4], radiation detection (RD) [5], and infrared thermography monitoring (IRT) [6] can achieve a wide range of detection in concrete structures. In the sonic technique, acoustic parameters of sound waves correlate with the state of the propagation medium; among them, acoustic emission (AE) is a passively received acoustic wave method tested during damage development [5,6,7], while ultrasonic pulse velocity (UPV) detects the medium’s state by calculating the wave speed of a direct wave [5,8].

In the last two decades, coda wave technology has been rapidly developing. Coda waves develop after multiple scattering superpositions in a medium, and they are extremely sensitive to changes in the state of a medium [9]. Concrete, as a strong scattering medium, allows monitoring of concrete structural conditions by analyzing changes in the acoustic parameters of the coda wave before and after perturbations. In the development of coda wave technology, improvement of the signal processing method is the key and the foundation.

Snieder et al. [10] proposed coda wave interferometry (CWI), which seeks to study the mapping relationship between the wave speed and the medium state by calculating the time shift between the wave trains before and after interference within the time window, as shown in Equations (1) and (2).

(1) $$CC\left(\tau \right)=\frac{\int_{k_{1}}^{k_{2}}\varphi^{\prime }\left(k\right)\varphi \left(k+\tau \right)dk}{\sqrt{\int_{k_{1}}^{k_{2}}{\varphi^{\prime }}^{2}\left(k\right)dk\int_{k_{1}}^{k_{2}}\varphi^{2}\left(k+\tau \right)dk}}$$

(2) $$\frac{\delta v}{v}=\frac{\tau }{k_{1}}$$

where $\varphi^{\prime }$ and φ denote the waveform in the initial and final state, ($k_{1}$, $k_{2}$) is the time window of the coda wave, τ represents the time shift, and δv/v represents the rate of wave speed change. In contrast to the calculation within the time window, Lobkis et al. [11] proposed the waveform stretching method (stretching) for the entire (or full) waveform to participate in the wave speed calculation. The above two methods are the mainstream methods for coda signal processing, and subsequent research has made improvements at different levels, such as improving the calculation efficiency through optimization algorithms [12] and introducing nonlinear-CWI, which needs to be carried out under stimulation [13], but essentially still enables the calculation of the wave speed. In addition, research on characteristics such as frequency and waveform has been carried out, but this still involves only a single feature. Niederleithinger et al. [14] proposed characterizing the similarity between waveforms through the square correlation coefficient (R^2^). Hafiz et al. [15] proposed coda wave comparison (CWC), which involves calculations of the correlation of the signal frequency spectra, and they showed that multiple scattering leads to time shift, frequency, and amplitude changes in the coda wave. In addition, with the rapid development of artificial intelligence (AI), methods of extracting signal features through convolutional neural networks (CNN) and associating them with output parameters have also begun to be applied in the processing of coda wave signals [16], and the results can be visualized through clustering.

While the development of coda wave signal processing methods has been accompanied by validation work in laboratories [16,17,18,19,20,21,22] as well as on real bridges [23,24], demonstrating the high sensitivity and computational accuracy of coda waves, their sensitivity also makes them susceptible to noise interference, which makes excessively large dispersion of the test results, making it difficult for engineers to accurately determine the true state of a structure. Under controlled temperature conditions, Niederleithinger et al. [25] found that the identified temperature values still exhibited abrupt changes, which they attributed to humidity variations. Wang et al. [24], in temperature detection on real bridges, demonstrated that environmental noise had a significant impact on the results’ accuracy and long-term detection was difficult. Most validation work aiming to mitigate the effects of environmental noise have only been conducted over short durations [15,16].

Multiple coda wave features are strongly correlated with the medium’s state, and in ultrasonic technology, it has been proven that using multi-parameter for comprehensive inversion can improve the anti-interference quality during identification [26,27]. Therefore, the authors propose sequentially extracting the time shift, the time stretch, and amplitude variation from the wave trains within the time window to obtain richer information about the medium’s state. By then combining these features with appropriate weights, a multi-feature combinatorial quantification model can be developed to reduce the standard deviation of the identification errors. Furthermore, under the influence of environmental temperature, the feasibility and effectiveness of the method are verified through ultrasonic testing on concrete I-beams for temperature detection.

## 2. Multi-Feature Decoupling and Combinatorial Quantization Methods

**Figure 1:**
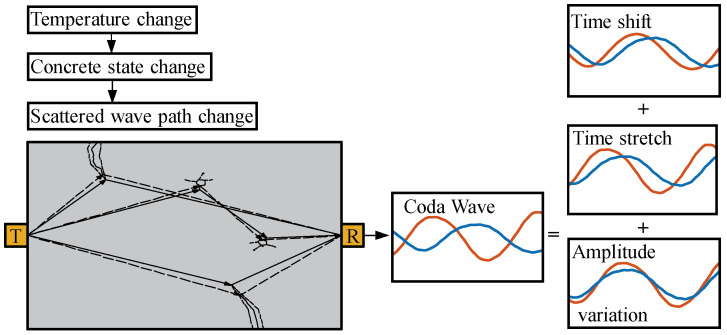
The multi-features of the scattered waves (the time shift, the time stretch, and the amplitude variation). The red and blue lines represent the coda waveforms formed by scattering in different temperature states of the medium.

A coda wave is a superposition of scattered waves from different propagation paths [9], as shown in Figure 1. Changes in the properties of the medium can cause changes in the state of micro-cracks in concrete, thereby causing changes in the propagation path [28,29]. On the different paths, the absorption attenuation and scattering attenuation of ultrasonic waves are different, so the superimposed coda waves show changes in the initial wave speed, the wave speed at each moment, and the amplitude [9,15], as shown in Figure 1. Previous single-parameter methods, such as the time window shift method [10], are based on the assumption that the relative speed changes between wave trains are uniform, and the amplitude variations are ignored. The stretching method [11] is based on the assumption of ignoring the amplitude variations during wave speed feature extraction. The method proposed in the following section sequentially extracts the time shift, the time stretch, and the amplitude variations of the wave trains within the time window.

### 2.1. Feature Extraction

(1) First, extract the time features by calculating the dual-parameter cross-correlation function, and take the time shift and stretching factor corresponding to its maximum value. This step combines the time window shift method [10] with the stretching method [11]. However, compared to the time window shift method, which requires a smaller time window to highlight the changes in wave speed characteristics, and the stretching method, which can cause the masking of coda wave signals when the direct wave energy is too large during entire (or full) waveform calculations, this method avoids the selection of windows and can obtain more feature changes in long sound and larger time windows. The formula is as follows:

(3) $$CC_{\max}\left(\tau ,\varepsilon \right)=\frac{\int_{k_{1}}^{k_{2}}\varphi^{\prime }\left(k\right)\varphi \left(\left(1+\varepsilon \right)k+\tau \right)dk}{\sqrt{\int_{k_{1}}^{k_{2}}{\varphi^{\prime }}^{2}\left(k\right)dk\int_{k_{1}}^{k_{2}}\varphi^{2}\left(\left(1+\varepsilon \right)k+\tau \right)dk}}$$

where ε represents the stretching factor, and $CC_{\max}$ corresponds to the maximum value of the cross-correlation function obtained under variations of τ and ε within the time window.

(2) After the previous operation is completed, the signal is denoted as $\varphi^{*}$, which is consistent on the time axis. At this time, the difference between $\varphi^{*}$ and the reference signal $\varphi^{\prime }$ manifests as a variation in amplitude. The amplitude of the signal is regarded as a vector in high-dimensional space, and vector rotation is performed in high-dimensional space to compensate for the variation in amplitude. Calculate the angle of vector rotation to characterize the variation in amplitude. First, we need to obtain the deflection direction vector $\alpha_{i}$ of each vector relative to the reference vector. Vector $\alpha_{i}$ is orthogonal to the reference vector. The formula is as follows:

(4) $$\alpha_{i}=\varphi^{*}-\varphi^{\prime }\cdot \left(\varphi^{\prime }\cdot \varphi^{*}\right)$$

The deflection direction of each vector relative to the reference vector is different; therefore, the deflection direction vector $\alpha_{i}$ also varies accordingly. To facilitate measurement, deflection is performed in the same deflection direction. The singular value decomposition (SVD) method is used to decompose the deflection direction matrix $D\left(\alpha_{1},\alpha_{2},\ldots \right)$, obtaining the direction with the greatest weight on each deflection direction, denoted as α.

(5) $$D=U\cdot \Sigma \cdot V^{T}$$

α is the left singular vector $U_{1}$, corresponding to the largest eigenvalue of the diagonal matrix Σ. At this time, the deflection angle in this direction can be calculated, which is the vector rotation angle θ.

(6) $$\theta =\arcsin\left(\varphi^{*}\cdot \alpha \right)$$

After the aforementioned two steps, the coda wave becomes consistent in terms of characteristics after undergoing time shift, stretching, and vector rotation. At the same time, three corresponding feature parameters are extracted, denoted as τ, ε, and θ.

### 2.2. Establishing the Temperature Identification Model

The temperature quantification relationship expressions $T_{\tau }(\tau )$, $T_{\varepsilon }(\varepsilon )$, and $T_{\theta }(\theta )$ are obtained through polynomial fitting, and the quantification errors of each quantification relationship are calculated. The size of the quantification error is key to whether the quantification relationship is accurately established. Here, by minimizing the quantification error of the model, the weight coefficients of the three quantification relationships are determined, denoted as $\gamma_{\tau }$, $\gamma_{\varepsilon }$, and $\gamma_{\theta }$, with their sum being 1. The root mean square error (RMSE) is used as the indicator, and the weight coefficient corresponding to its minimum value is taken.

(7) $$S_{\min}(\gamma_{\tau },\gamma_{\varepsilon },\gamma_{\theta })=\sqrt{\sum_{i=1}^{N}{\left(\left(\gamma_{\tau }T_{\tau ,i}+\gamma_{\varepsilon }T_{\varepsilon ,i}+\gamma_{\theta }T_{\theta ,i}\right)-T_{i}\right)}^{2}/N}$$

$S_{\min}$ is the minimum RMSE obtained when the weight coefficient changes, *N* is the sample size of the temperature set, and the weight coefficients corresponding to the three parameters at this time are ${\gamma^{\prime }}_{\tau }$, ${\gamma^{\prime }}_{\varepsilon }$, and ${\gamma^{\prime }}_{\theta }$. The final identification temperature $T_{d}$ is represented by the three identification temperatures corresponding to the combination of weight coefficients:

(8) $$T_{d}\left(\tau ,\varepsilon ,\theta \right)={\gamma^{\prime }}_{\tau }T_{\tau }\left(\tau \right)+{\gamma^{\prime }}_{\varepsilon }T_{\varepsilon }\left(\varepsilon \right)+{\gamma^{\prime }}_{\theta }T_{\theta }\left(\theta \right)$$

Based on the properties of variance and the assumption in regression analysis that the residuals of errors are i.i.d, it is easy to prove that after introducing a coefficient less than 1, the variance (degree of dispersion) of the combined error will decrease.

## 3. Laboratory Experiments

### 3.1. Specimens and Equipment

**Figure 2:**
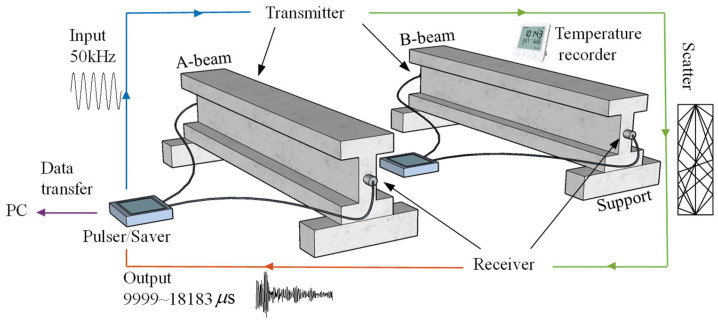
The pulser transmits a high-voltage pulse, which excites the transmitter and generates ultrasonic waves. The scattered ultrasonic waves are input into the receiver and recorded by the saver. The data in the saver are transmitted to the PC end for processing.

Two concrete I-beams with identical specifications and cast from the same batch are referred to as A and B. The specimen is 2 m long and 0.45 m high; the flange is 0.3 m wide and 0.1 m thick; and the web is 0.25 m high and 0.1 m thick. The concrete strength is C30, the fineness modulus of the fine aggregate is 2.7, and the coarse aggregate is crushed stone of 5~10 mm. The mass ratio is cement:fine aggregate:coarse aggregate:water:fly ash:slag = 1.00:2.50:3.85:0.55:0.22:0.16. Both the longitudinal reinforcement and the stirrup use A10 rebar, and the stirrup spacing is 0.15 m. The RSM-SY5(T) non-metal ultrasonic detector combines high-voltage pulse transmission with data recording and storage capabilities. The sampling length was 1024, the sampling interval was 8 μs, the trigger delay was 9999 μs, the pulse width was 5 μs, and the gain was 80 dB. The experiment utilized the JHP01 piezoelectric transducer, with a diameter of 40 mm and a central frequency of 50 kHz. An epoxy resin coupling agent was used to fix the transducer at the neutral axis position of the cross-section at both ends of the beam; one end transmits and the other end receives. The chosen frequency was based on previous research to ensure sufficient scattering [9,14], excellent signal-to-noise ratio (SNR), limited attenuation, and high sensitivity as well as resolution for detecting medium variations. Fixing the transducer is beneficial to obtain a high SNR and stable signal [25]. The YEM-70L automatic temperature recorder was used, positioned near the concrete beam. It collected samples at 5 min intervals, with a resolution of 0.1 °C. The test system is shown in Figure 2.

### 3.2. Data Collection Scheme

The experiment synchronously collected coda wave signals and their corresponding environmental temperature values for two specimens under a natural environment (14~21 °C). The experiment was conducted in two phases, each phase lasting 10 days, for a total duration of 20 days. Each day, 14 groups of data were collected from 9:00 AM to 4:00 PM (a period when temperature variations are relatively stable). In each group (26 min), 400 repeated acquisitions were performed, resulting in a time domain signal of coda waves containing 1024 data points. Each group collected 5 environmental temperature values. The following section uses the data from the first phase to establish a temperature quantification relationship and the temperature quantification relationship to identify and verify the data from the second phase. The collection and processing methods of beam B were the same, and its results are used for comparison and analysis with beam A.

### 3.3. Data Preprocessing

For each group, the signals and temperature values were averaged to reduce experimental random errors. The signal resolution was enhanced from the original 8 μs (1024 data points) to 1 μs (8177 data points) using Fourier interpolation. The average value of the amplitude was subtracted from each sample point of the signals to make its mean value 0, and normalization was applied to ensure consistent energy levels across all signals. The impact of unstable transmitter energy was mitigated, and the signals were standardized to a common format to enhance feature performance. The size of the data before and after preprocessing is shown in Table 1.

**Figure 3:**
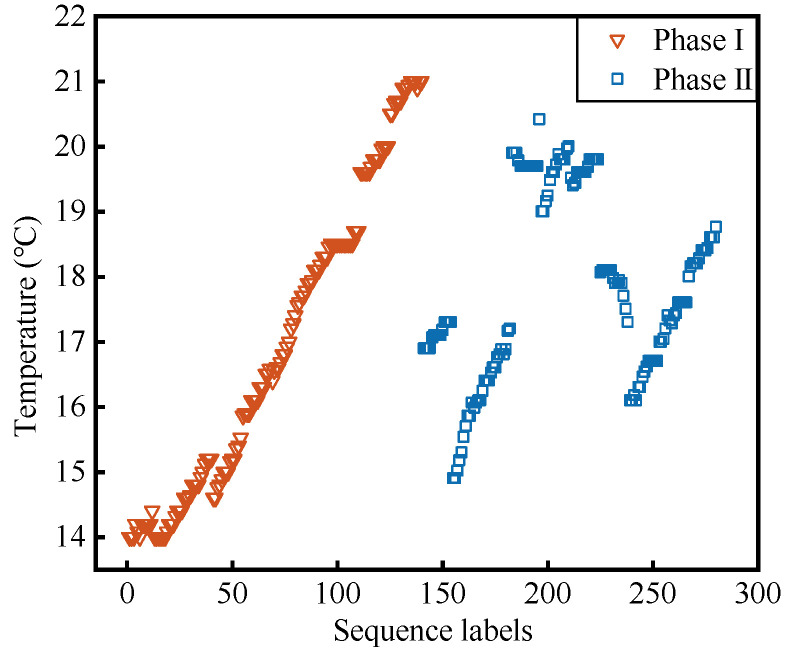
Temperature data were collected during the two phases of the test.

The first phase of the experiment involves a continuous heating process, while the second phase fluctuates within the range of temperature changes in the first phase, as shown in Figure 3.

## 4. Results

### 4.1. Combined Quantification Results

**Figure 4:**
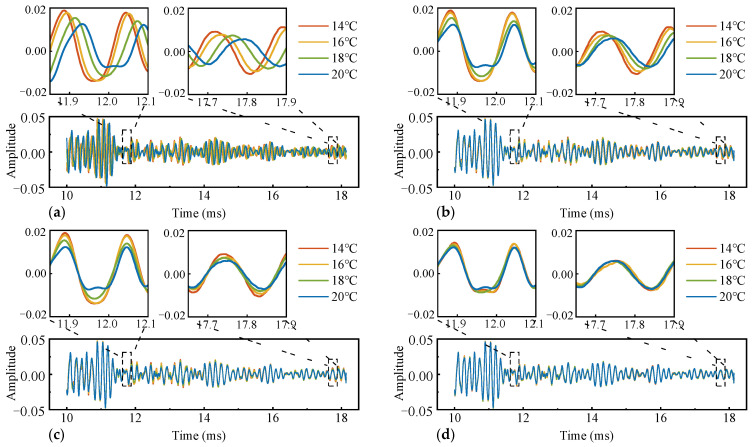
(**a**) The original waveform; (**b**) the waveform erased of the time shift; (**c**) the waveform erased of the time shift and the time stretch; (**d**) the waveform erased of the time shift, the time stretch, and the amplitude variation.

**Figure 5:**
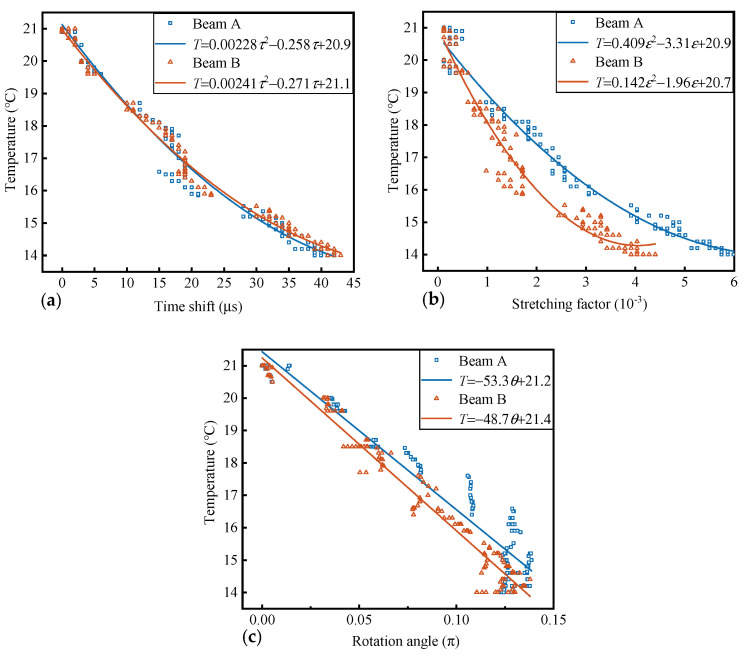
The relationship between the three parameters and temperature variations. (**a**) τ − *T*; (**b**) ε − *T*; and (**c**) θ − *T*.

From the first phase of the experiment, the signal corresponding to the highest temperature (21 °C) collected is used as the reference signal. Three parameters of the first-phase signal are extracted and mapped to the corresponding temperature values. As shown in Figure 4a, as the temperature rises, the waveform shows varying degrees of delay. In Figure 4b, after the time shift, the initial phase difference within the time window is compensated, and it shows a stretching trend as the temperature rises. In Figure 4c, after the stretching, the deviation on the time axis is compensated, and the difference between the waveforms is the variation in amplitude. In Figure 4d, after rotating in high-dimensional space, the waveforms at each temperature tend to be consistent. The average correlation coefficient (R) of signals in the signal set with the reference signal increases from 0.39 to 0.94, 0.97, and 0.99 in sequence. Each of the three parameters establishes a relationship with temperature variations through polynomial fitting. As shown Figure 5, the time shift and stretching factor decrease as a quadratic function with increasing temperature, while the vector rotation angle decreases as a linear function with increasing temperature.

The coefficient of determination of goodness-of-fit (R^2^) for the three quantification relationships all reach a level of 0.90. Rather than choosing one, their quantification ability can be enhanced through a weighted combination. According to the steps of combinatorial quantitative establishment, it can be found that when the weight coefficients are 0.62, 0.16, and 0.22, (beam B, 0.44, 0.37, 0.19, respectively), the RMSE of the combined relationship is minimized. Substituting these into the Equation (8) yields the temperature identification expression.

**Figure 6:**
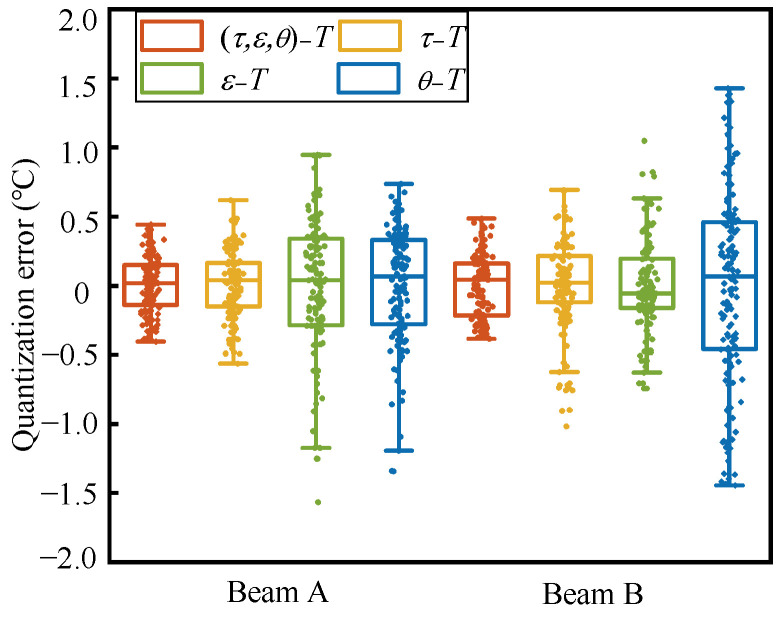
Box plots of errors for quantization relations established by feature parameter combinations and decoupled parameters.

RMSE, the standard deviation (SD) of absolute errors, and R^2^ are used as indicators to evaluate the magnitude of quantification error, the dispersion of error, and the degree of temperature characterization by the parameters. The RMSE of the combined quantification relationship is 0.21 °C, which is 16%, 57%, and 50% lower than the quantification relationships independently established by the three feature parameters after decoupling, respectively. The SD is 0.12 °C, which is 15%, 60%, and 52% lower, respectively, and R^2^ is elevated to a level of 0.99 (in beam B, the RMSE is 0.22 °C, reduced by 32%, 36%, and 69%; the SD is 0.11 °C, reduced by 50%, 50%, and 74%; and the R^2^ is elevated to a level of 0.99). As shown in Figure 6, the line in the middle of the box in the box plot represents the median value of the sample quantization error set, denoted as Q2. The upper and lower edges of the box represent the values of the errors at 25% and 75%, respectively, denoted as Q1 and Q3. The interquartile range (IQR) is denoted as Q3–Q1. The two short lines extending outward represent Q1 − 1.5IQR and Q3 + 1.5IQR, respectively. Data outside these two lines are considered outliers. The errors of τ − *T*, ε − *T*, and θ − *T* are all within the ±(0.5~1.5) °C range. In the data of beam A, ε − *T* and θ − *T* both showed outliers. In beam B, τ − *T* and ε − *T* both showed outliers. Compared to the relationship established for a single parameter with temperature variations, the dispersion of the errors from the combined relationship between the three parameters and the temperature variations decreases and there are no outliers. This is consistent with the results presented by the calculated RMSE and SD above. In summary, it is feasible to mutually map the changes in some properties of the medium through the time shift, the time stretch, and vector rotation angle between the coda waves. In this study, there is a good mapping relationship with temperature, and by combining them through weight coefficients, the quantification error can be made smaller and more stable, and a more accurate quantification relationship can be obtained to provide higher precision and stability identification.

### 4.2. Quantification Results Established by a Single Parameter

The above analysis has discussed the improvement of the combined quantification relationship compared to the quantification relationships established separately by the three feature parameters after decoupling. This section will conduct a comparative analysis under the condition of feature coupling: that is, only one of the three transformations is performed. The stretching method involves the overall stretching of the entire waveform, which requires no initial phase difference between the waveforms within the time window. Moreover, according to the determination of the weight coefficients above, after the extraction of the first two features, the quantification relationship established by the vector rotation angle still has weight allocation when combined. This indicates that even if the initial phase is aligned and then the waveform is stretched or compressed, the weight of the established quantification relationship cannot be one. Therefore, this section does not analyze it.

**Figure 7:**
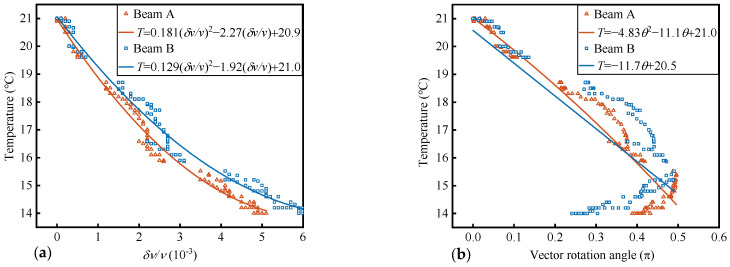
The relationship between independent feature parameters and temperature. (**a**) δv/v − *T*, δv/v by CWI; and (**b**) θ − *T*, θ by SVD.

Using the same reference signal, the δv/v and θ of the first phase signal are independently extracted, and their respective temperature quantification relationships are established; the method of extracting θ is called SVD. As shown in Figure 7a, the trend of δv/v changing with temperature is similar to τ. The difference is due to the simultaneous determination of τ and ε in this paper. As shown in Figure 7b, the θ in the direction of the largest deflection increases as the temperature decreases. When the temperature change exceeds about 6 °C, the trend of θ change reverses. The reason is that as the temperature change increases, the signal difference increases, causing the angle with the reference vector to exceed 90°, resulting in the reverse change of the θ, indicating that the quantification interval of this method will be limited. In the operation of this paper, with the extraction of the first two features, the difference between signals is greatly reduced, so that the quantification interval of the third parameter is increased. For the τ and ε of the coda wave, many factors such as stress and temperature will cause structural deformation, leading to the closure or development of micro-cracks, thereby changing the scattering wave path. These influences are reflected in the first two features [17,25,30]. However, after extracting the first two features, multivariate linear regression processing in high-dimensional space may be used to utilize different influencing factors having different spatial deflection directions to achieve decoupling between different influencing factors. Study [30] also noted the need to separate different influencing factors.

**Figure 8:**
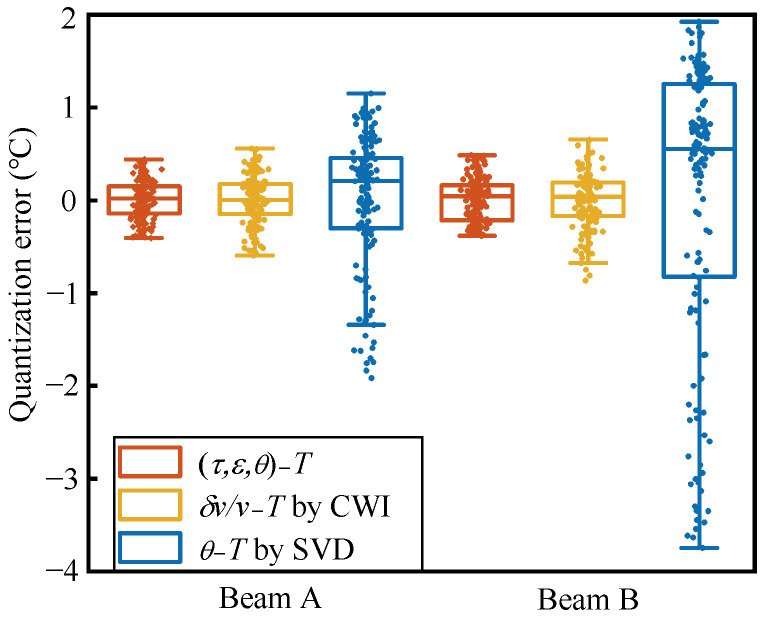
Error box plots of the relationship established between the parameters of the independent features and the temperature variations, and the relationship established between the combination of the three parameters and the temperature variations.

Compared to quantitative relationships established by a single parameter, the RMSE is reduced by 19% and 71% respectively, the SD is reduced by 25% and 73%, and the R^2^ is increased from 0.93 and 0.88 to a level of 0.99 (beam B, the RMSE is increased by 24%, 86%; the SD is increased by 40%, 76%; and the R^2^ is increased from 0.93 and 0.53 to a level of 0.99). The experiment in this paper was conducted in a natural environment. Temperature is the main factor affecting signal fluctuations in the natural environment. Compared to temperature, the magnitude of the impact of humidity and other noise on coda wave characteristics can be ignored [18,19]. However, it still affects the stability and repeatability of the coda wave signal to a certain extent. This causes interference in the establishment of quantitative relationships; however, the comparisons of the methods in this paper use data under the same environmental impacts. This indicates that the combined quantified relationship of feature parameters can mitigate the impact of noise or outliers on the quantified relationship of individual parameters, resulting in a reduced SD of quantification errors. By extracting more features, more medium state information is obtained, and the average correlation coefficient (R) with the reference signal is increased from 0.93 and 0.85 to 0.99. As shown in Figure 8, compared to the relationship established by only one parameter with temperature variations, the dispersion of the errors of combined quantization method is the smallest. The dispersion of the errors in δv/v − *T* is relatively close to the combinatorial quantization, but both the δv/v − *T* and the θ − *T* have outliers in their errors. The θ − *T* is limited by the size of the quantization interval, leading to poor results.

### 4.3. Identification Result

**Figure 9:**
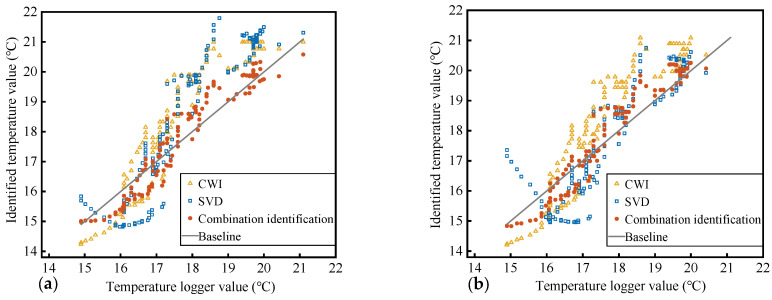
(**a**) The combinatorial identification, CWI, and SVD identification results of beam A. (**b**) Identification results of beam B.

In the process of monitoring or identifying the state of the medium, the reproducibility and stability of the identification results are key to the practical application of coda wave technology. This not only requires the accuracy of the quantification relationship established in the above discussion but also requires that the signals collected afterward during identification still satisfy the established mapping relationship. The combined quantification relationship established above is verified by the signal and temperature data collected in the second phase. The identification result of beam A is shown in Figure 9a; independent of the data of beam A, the quantification relationship is established and the identification verification is carried out for the data of beam B, and the result is shown in Figure 9b. The identification accuracy is evaluated by mean absolute error (MAE), and the identification stability is evaluated by the SD of absolute error. The results are shown in Table 2. The identification accuracy of the combined quantification is improved by 60~70%, and the stability is improved by 50~60%. Among the 140 items of temperature identification data, the identification results of the combined quantification are closer to the temperature recorder readings for 126 and 125 data items, accounting for 89% and 90%, respectively, compared to the identification results of CWI and SVD (beam B, 97 and 94 data items are closer, accounting for 69% and 67%, respectively).

**Figure 10:**
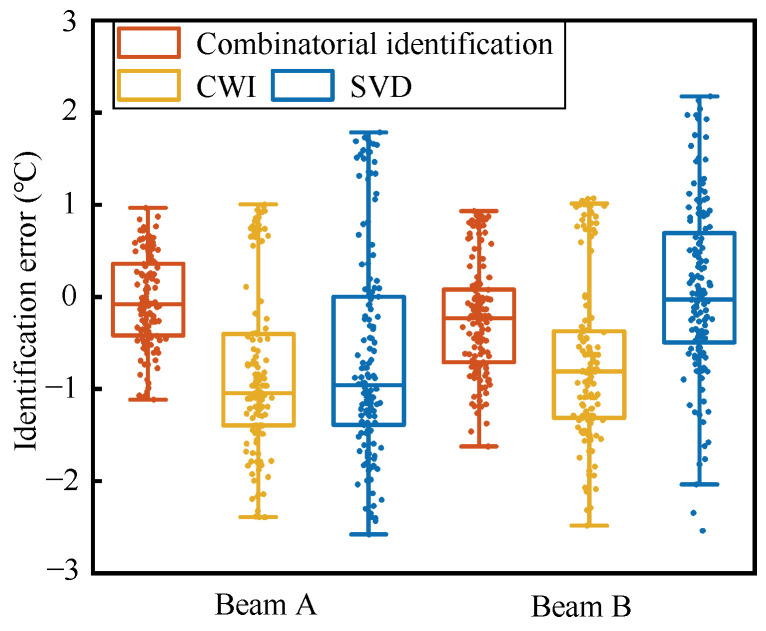
Identification errors box plot of different methods.

Similarly to the decrease in the degree of dispersion of the quantification error, during identification, under the influence of noise and abnormal signals, having only one parameter as the input value can lead to a large deviation in the results obtained; by combining the temperature identification results corresponding to the three feature parameters of the collected signal, the interference to the identification result is weakened to a certain extent, providing a relatively accurate and stable evaluation of the structural state. The results obtained from the two specimens in this experiment are almost consistent. The temperature identification values of the three methods shown in Figure 9 have similar trends, and there is a certain deviation from the trend of the true value of temperature. This is caused by the “Kaiser effect” [23]: that is, the memory effect of the medium. The temperature change in the second phase will not cause the concrete to develop new micro-cracks, causing the mapping relationship between the features and the temperature to change; this is consistent with the conclusions of the study [24,28]. As shown in Figure 10, except for the identification results of the SVD method for the beam B data, the medians of the identification errors of the rest of CWI and SVD deviate from 0 °C and are close to −1 °C. The errors of the CWI and SVD methods are unevenly distributed on both sides of 0 °C, and the influence of the “Kaiser effect” is more obvious. In comparison, the dispersion of the errors of the combined identification results is the smallest, and the median of the errors is closer to 0 °C. The errors for the combinatorial identification method are more evenly distributed on both sides of 0 °C, indicating that the combined identification can reduce the impact of this effect to a certain extent.

## 5. Conclusions

(1) The multi-features of the ultrasonic waves can be extracted by the time shift, the time stretch, and amplitude variation of the wave trains within the time window, and these three parameters can be used to establish a good function relationship with the temperature variations of the beams. Based on the three functions, the minimum error weight combination of three parameters can be obtained through a multivariate function weighting calculation. After the combination, the quantization function R^2^ can reach 0.99.

(2) In the process of SHM with noise interference, the single-feature method establishes a relationship between the medium’s state and one parameter, and the results of identification will exhibit a large discreteness; compared with the single-feature method, the results of the combinatorial quantization method based on the multi-features give a more stable assessment of the structural states. The order of magnitude of the identification accuracy is 10^−1^ °C, and the accuracy and stability are both improved by more than 50%.

## Figures and Tables

**Table 1:** The size of raw data and preprocessed data.

	Raw Data	Preprocessed Data
Signal set	1024 (points/8 μs) × 400 (times) × 14 (groups) × 10 (days) × 2 (phases)	8177 (points/1 μs) × 140 (items) × 2 (phases)
Temperature set	5 (times) × 14 (groups) × 10 (days) × 2 (phases)	140 (items) × 2 (phases)

**Table 2:** Evaluation of identification results of different methods for two specimens.

Specimen	Methodologies	MAE (°C)	The SD of Absolute Error (°C)
Beam A	CWI	1.12	0.51
	SVD	1.15	0.64
	Combinatorial identification	0.40	0.26
Beam B	CWI	1.03	0.48
	SVD	0.74	0.56
	Combinatorial identification	0.38	0.28

## Data Availability

All data generated or analyzed during this study are included in this article. All data included in this study are available upon request by contact with the corresponding author.

## References

[1] Kishida K., Imai M., Kawabata J., Guzik A. (2022). Distributed Optical Fiber Sensors for Monitoring of Civil Engineering Structures. Sensors.

[2] Na W.S., Baek J. (2018). A Review of the Piezoelectric Electromechanical Impedance Based Structural Health Monitoring Technique for Engineering Structures. Sensors.

[3] Yang M., Zhang H., Ma X.T., Zheng Y., Zhou J.T. (2024). Quantitative detection of rebar corrosion by magnetic memory based on first-principles. Eng. Res. Express.

[4] Boldrin P., Fornasari G., Rizzo E. (2024). Review of Ground Penetrating Radar Applications for Bridge Infrastructures. NDT.

[5] Zheng Y.X., Wang S.Q., Zhang P., Xu T.X., Zhuo J.B. (2022). Application of Nondestructive Testing Technology in Quality Evaluation of Plain Concrete and RC Structures in Bridge Engineering: A Review. Buildings.

[6] Zhang J.H., Peng L.H., Wen S.Z., Huang S.L. (2024). A Review on Concrete Structural Properties and Damage Evolution Monitoring Techniques. Sensors.

[7] Han Q.H., Xu J., Carpinteri A., Lacidogna G. (2015). Localization of acoustic emission sources in structural health monitoring of masonry bridge. Struct. Control Health Monit..

[8] Bogas A.J., Gomes G.M., Gomes A. (2013). Compressive strength evaluation of structural lightweight concrete by non-destructive ultrasonic pulse velocity method. Ultrasonics.

[9] Planes T., Larose E. (2013). A review of ultrasonic coda wave interferometry in concrete. Cem. Concr. Res..

[10] Snieder R., Gret A., Douma H., Scales J. (2002). Coda wave interferometry for estimating nonlinear behavior in seismic velocity. Science.

[11] Lobkis O.I., Weaver R.L. (2003). Coda-wave interferometry in finite solids: Recovery of P-to-S conversion rates in an elastodynamic billiard. Phys. Rev. Lett..

[12] Liu S., Zhu J., Wu Z. (2015). Implementation of coda wave interferometry using Taylor series expansion. J. Nondestruct. Eval..

[13] Jean-Baptiste L., Zhang Y., Odile A., Olivier D., Vincent T. (2017). Evaluation of crack status in a meter-size concrete structure using the ultrasonic nonlinear coda wave interferometry. J. Acoust. Soc. Am..

[14] Niederleithinger E., Wolf J., Mielentz F., Wiggenhauser H.E., Pirskawetz S. (2015). Ultrasonic Transducers for Active and Passive Concrete Monitoring. Sensors.

[15] Hafiz A., Schumacher T. (2018). Monitoring of stresses in concrete using ultrasonic coda wave comparison technique. J. Nondestruct. Eval..

[16] Saenger E.H., Finger C., Karimpouli S., Tahmasebi P. (2021). Single-Station Coda Wave Interferometry: A Feasibility Study Using Machine Learning. Materials.

[17] Larose E., Hall S. (2009). Monitoring stress related velocity variation in concrete with a 2 × 10^−5^ resolution using diffuse ultrasound. J. Acoust. Soc. Am..

[18] Larose E., Rosny J.D., Margerin L., Anache D., Gouedard P., Campillo M., Tiggelen V.B. (2006). Observation of multiple scattering of kHz vibrations in a concrete structure and application to monitoring weak changes. Phys. Rev. E.

[19] Payan C., Garnier V., Moysan J. (2009). Effect of water saturation and porosity on the nonlinear elastic response of concrete. Cem. Concr. Res..

[20] Gret A., Snieder R., Scales J. (2006). Time-lapse monitoring of rock properties with coda wave interferometry. J. Geophys. Res..

[21] Payan C., Garnier V., Moysan J., Johnson P.A. (2008). Determination of nonlinear elastic constants and stress monitoring in concrete by coda waves analysis. Proc. Meet. Acoust..

[22] Moradi-Marani F., Kodjo A.S., Rivard P., Lamarche C.P. (2014). Effect of the Temperature on the Nonlinear Acoustic Behavior of Reinforced Concrete Using Dynamic Acoustoelastic Method of Time Shift. J. Nondestruct. Eval..

[23] Sthler S.C., Sens-Schnfelder C., Niederleithinger E. (2011). Monitoring stress changes in a concrete bridge with coda wave interferometry. J. Acoust. Soc. Am..

[24] Wang X., Chakraborty J., Niederleithinger E. (2021). Noise reduction for improvement of ultrasonic monitoring using coda wave interferometry on a real bridge. J. Nondestruct. Eval..

[25] Niederleithinger E., Wunderlich C. (2013). Influence of small temperature variations on the ultrasonic velocity in concrete. AIP Conf. Proc..

[26] Zhang W.X. (2021). Acoustic multi-parameter full waveform inversion based on the wavelet method. Inverse Probl. Sci. Eng..

[27] Niu Z., Wang W., Huang X., Lai J. (2021). Integrated assessment of concrete structure using Bayesian theory and ultrasound tomography. Constr. Build. Mater..

[28] Bompan K.F., Haach V.G. (2018). Ultrasonic tests in the evaluation of the stress level in concrete prisms based on the acoustoelasticity. Constr. Build. Mater..

[29] Ma B., Liu S., Ma Z., Wang Q.-A., Yu Z. (2022). Numerical Parametric Study of Coda Wave Interferometry Sensitivity to Microcrack Change in a Multiple Scattering Medium. Materials.

[30] Diewald F., Epple N., Kraenkel T., Gehlen C., Niederleithinger E. (2022). Impact of External Mechanical Loads on Coda Waves in Concrete. Materials.

